# Association between single-nucleotide polymorphisms in miRNA and breast cancer risk: an updated review

**DOI:** 10.1186/s40659-021-00349-z

**Published:** 2021-08-28

**Authors:** Trinidad Arancibia, Sebastian Morales-Pison, Edio Maldonado, Lilian Jara

**Affiliations:** 1 Programa de Genética Humana, Instituto de Ciencia Biomédicas (ICBM), Facultad de Medicina, Universidad de Chile, 8380453 Santiago, Chile; 2 Programa Biología Celular y Molecular, Facultad de Medicina, Universidad de Chile, 8380453 Santiago, Chile

**Keywords:** Polymorphisms, miRNAs, Breast cancer risk, Association studies

## Abstract

Breast cancer (BC), a heterogeneous, aggressive illness with high mortality, is essentially a genomic disease. While the high-penetrance genes *BRCA1* and *BRCA2* play important roles in tumorigenesis, moderate- and low-penetrance genes are also involved. Single-nucleotide polymorphisms (SNPs) in microRNA (miRNA) genes have recently been identified as BC risk factors. miRNA genes are currently classified as low-penetrance. SNPs are the most common variations in the human genome. While the role of miRNA SNPs in BC susceptibility has been studied extensively, results have been inconsistent. This review analyzes the results of association studies between miRNA SNPs and BC risk from countries around the world. We conclude that: (a) By continent, the largest proportion of studies to date were conducted in Asia (65.0 %) and the smallest proportion in Africa (1.8 %); (b) Association studies have been completed for 67 different SNPs; (c) 146a, 196a2, 499, 27a, and 423 are the most-studied miRNAs; (d) The SNPs rs2910164 (miRNA-146a), rs11614913 (miRNA-196a2), rs3746444 (miRNA-499) and rs6505162 (miRNA-423) were the most widely associated with increased BC risk; (e) The majority of studies had small samples, which may affect the precision and power of the results; and (f) The effect of an SNP on BC risk depends on the ethnicity of the population. This review also discusses potential explanations for controversial findings.

## Introduction

Breast cancer (BC) has a high mortality rate and is the most common type of cancer among women worldwide. The disease is characterized by expression of aberrant genes that confer tumors with heterogeneous morphology and aggressiveness, producing diverse clinical manifestations [1, 2]. BC susceptibility genes and variants are currently classified into three categories that reflect the probability of developing the disease (high, moderate, or low penetrance) [3]. The most common and well-known high-penetrance susceptibility genes, *BRCA1* and *BRCA2*, account for only about 16 % of cases. There is consensus that moderate- and low-penetrance genes are likely responsible for a significant percentage of familial BC in *BRCA1/2*-negative families [4]. Recent findings suggest that microRNAs (miRNAs) are low-penetrance genes [5]. miRNAs are small, non-coding, single-stranded RNAs approximately 18–25 nucleotides in length [6]. These molecules have drawn the attention of researchers given their numerous roles in cellular, physiological, and pathological processes. miRNAs regulate gene expression by degrading or blocking translation of targets [7, 8] and are specific to different mRNAs. Approximately 30 % of all human genes are regulated by miRNAs [9, 10]. Current data supports the assertion that these RNAs play important and diverse roles in many molecular pathways and biological processes, including development, apoptosis, differentiation, and cell proliferation [11, 12]. Furthermore, miRNAs have been implicated in various human diseases, including cancer. Genome-wide miRNA expression profiling studies have demonstrated that almost all cancer types show specific profiles of up- and downregulated miRNAs [13, 14]. Growing evidence also indicates that miRNAs can function both as oncogenes and tumor suppressors [15, 16]. In 2005, Iorio et al. [17] described an association between miRNAs and BC for the first time, and evidence of their contribution to disease etiology has mounted in the 16 years since their discovery. Several environmental and genetic elements are involved in the various types of BC, and genetic variations in tumor-suppressor and oncogenes are associated with carcinogenesis [18].

Single-nucleotide polymorphisms (SNPs) are the most common form of variation present in the human genome. SNPs in miRNA regions can alter expression of the gene, provoke aberrant maturation, and alter target-binding affinity and specificity [19]. Many epidemiological studies have examined the association between SNPs in miRNAs and cancer [20], concluding that some of these polymorphisms contribute to BC susceptibility in different populations. Research on miRNA genes is critical for understanding the biology of breast tumors, developing new diagnostic strategies, and identifying more effective therapies [21].

SNPs are ethnicity-specific; as a result, findings for a specific population are not always applicable to other groups. Moreover, many countries have several ancestral lineages. Therefore, we conducted an extensive literature review to clarify the wealth of findings on this important topic in the international context. This review discusses the implications of the many association studies between miRNA genetic variations (SNPs) and BC susceptibility published between 2009 and 2020.

### The landscape of breast cancer predisposition: past and present

Risk factors for BC include gender, age, hormonal factors, and, most significantly, genetic predisposition (family history). Characteristics of genetic predisposition include dominant autosomal inheritance, high penetrance (that is, a carrier has a 67 % risk of developing BC by 70 years of age and an 80 % risk by 80 years), a genetic frequency of 0.003, and a carrier frequency of 0.006 [22]. The data suggest that 1 in 20 women with BC, and 1 in 200 women in the general population, carry a genetic predisposition, making BC one of the most widely-distributed heritable pathologies. The existence of a gene or genes responsible for a heritable predisposition to breast and ovarian cancer was suggested more than a century ago [23] and has been supported by a large quantity of epidemiological literature over the past 80 years [22, 24–28]. Segregation studies have indicated the existence of one or more genes that determine predisposition for BC.

The discovery of the tumor-suppressor genes *BRCA1* (MIM 113,705) [29] and *BRCA2* (MIM 600,185) was a major advance in elucidating the genetic etiology of BC [30, 31]. *BRCA1/2* are considered high-penetrance BC susceptibility genes [32, 33]. As noted above, the literature indicates that mutations in *BRCA1/2* are responsible for an average of 16–20 % of the risk for hereditary BC [3, 34, 35]. However, genome-wide linkage analyses using large samples of *BRCA1/2*-negative families have failed to map additional high-penetrance susceptibility loci [36]. It is likely, therefore, that moderate- and low-penetrance genes are responsible for a significant percentage of cases in *BRCA1/2*-negative families [4]. These low-penetrance genes include miRNAs [5].

### General features of miRNAs and their relationship with cancer

As mentioned above, miRNAs are small, non-coding, single-stranded RNAs that have drawn the attention of researchers given their roles in many biological processes [6]. miRNAs regulate gene expression mainly by binding to the 3’-UTR of the target mRNA [7, 8]. However, some studies have reported that miRNAs can also bind to the 5’-UTR [20, 37]. It has been proposed that, depending on the base pairing between the miRNA and target, the negative regulatory effect could vary from weak repression of protein translation to complete cleavage of the mRNA [38]. Since their initial discovery in *C. elegans* by Lee et al. (1993) [7], more than 1200 miRNAs have been identified in humans, although the specific functions of most remain unknown [39]. A better understanding of how miRNAs regulate their targets would likely yield a great deal of insight into the genetic complexity underlying human health and disease [10]. Many miRNAs have already been implicated in various human diseases such as cardiovascular pathologies, psychiatric disorders, neurodegenerative conditions, and cancers [10]. There is increasing evidence for a vital role of aberrant miRNA expression in the complex and multistep process of carcinogenesis, with miRNA genes acting both as tumor suppressors and oncogenes [40]. As cancer is the second-leading cause of death worldwide [41], understanding its pathogenesis is critical; delineating the role of miRNA in this process would be extremely helpful. One of the first direct links between miRNA and cancer was reported by Callin et al. [42], who found decreased miR-15a and miR-16-1 levels in patients with chronic lymphocytic leukemia. In solid tumors, Michael et al. (2003) [43], identified 28 miRNAs that were differentially expressed in colonic adenocarcinoma vs. normal mucosal tissue, reporting that miR-143 and miR-145 levels were significantly lower in tumors than normal tissues.

As noted, nearly all cancer types have specific profiles of up- and downregulated miRNAs [13, 14]. Several studies have described specific miRNA expression signatures in breast carcinomas [17], primary glioblastomas [44], hepatocellular carcinomas [45], papillary thyroid carcinomas [46], and lung cancer [47]. A large profiling analysis of 540 samples from solid tumors in the lung, breast, stomach, prostate, colon, and pancreas demonstrated that 43 miRNAs were deregulated compared to matched normal tissues [48].

miRNAs can likely function as oncogenes when their targets are onco-suppressor molecules and as tumor-suppressor genes when their targets are oncogenes [15, 16]. Furthermore, a miRNA can function as both a tumor-suppressor and an oncogene depending on the cancer type and cellular context [49]. In fact, a duality of function in different types of cancers has been reported for many miRNAs. One example is miR-125b, which plays opposite roles in different cancer types and cell lines. As a tumor suppressor, miR-125b is downregulated in ovarian, thyroid, breast, and oral squamous-cell carcinomas, promoting cell proliferation and cell-cycle progression [50]. On the other hand, miR-125b is an oncogene in prostate cancer, glioblastomas, and neuroblastomas. miR-125b inhibits apoptosis in a p53-dependent manner in neuroblastoma cells and promotes cell proliferation and invasion in prostate cancer cells [51, 52]. After early studies suggested a role for miRNA genes in the pathogenesis of human cancers, platforms were developed to assess global miRNA expression. The goal of these analyses was to assess the potential of miRNAs in tumor classification and as diagnostic, predictive, or prognostic biomarkers [12].

### miRNAs and breast cancer

Microarrays containing all known human miRNAs can be used to identify miRNAs that are differentially expressed in normal and tumor samples, and this approach may be used to determine which miRNA molecules are involved in human cancer. In BC, miRNA microarrays have been used to evaluate miRNA expression profiles in 10 normal and 76 neoplastic breast tissues, identifying 29 miRNAs whose expression was significantly deregulated (p < 0.05) and a smaller set of 15 miRNAs that were able to predict whether a sample was tumor or normal breast tissue with 100 % accuracy [17, 53]. Among the differentially-expressed miRNAs, miR-10b, miR-125b, miR-145, miR-21 and miR-155 were the most consistently deregulated in BC. miR-10b, miR-125b and miR-145 were downregulated, while miR-21 and miR-155 were up-regulated, suggesting that they may act as tumor-suppressor or oncogenes, respectively. In addition, it was possible to identify miRNAs whose expression was correlated with specific BC histopathologic features, such as estrogen and progesterone receptor expression (miR-30), lymph node metastasis (let-7f-1, let-7a-3, let-7a-2) or high proliferative index (let-7c, let-7d) in tumor samples. Therefore, several expression profiling studies have demonstrated that there is a large number of deregulated miRNAs in human BC.

### Association studies between miRNA SNPs and breast cancer susceptibility

We conducted a literature review of association studies between miRNA genetic variations (SNPs) and BC susceptibility. PubMed, EBSCO, SciELO, and Google Scholar databases were searched for all studies involving SNPs in miRNAs related to BC risk around the world. The search terms included: “SNPs in miRNA and breast cancer susceptibility;” “association of SNPs in miRNAs with breast cancer risk;” “South America;” “North America;” “Latin America;” “Europe;” “Asia;” “Oceania;” and other terms associated with different countries. Manuscripts published between the years 2009 and 2020 were considered. Only papers published in English were reviewed. Non-human studies, in vitro or in vivo studies, and studies focused on topics other than SNPs in miRNAs and BC susceptibility were excluded. Inclusion criteria were: (a) association studies between SNPs in miRNAs and BC susceptibility; (b) country of origin for BC cases was specified; (c) the miRNAs and SNPs studied were identified. After the search was completed, studies were organized in a Google Sheets spreadsheet. Out of a total of 72 studies, 15 studies were removed due to lack of information regarding the inclusion criteria and 57 were included in this review.

Association studies were found in 17 countries (Australia, Brazil, Chile, China, France, Germany, India, Iran, Ireland, Israel, Italy, Pakistan, Saudi Arabia, Spain, Tunisia, USA, and Vietnam) on 5 continents. Of the 57 association studies included in this review, one was conducted in Africa (1.8 %), 37 in Asia (65.0 %), 9 in Europe (15.8 %), 3 in North America (5.3 %), 3 in Oceania (5.3 %), and 4 in South American countries (7.0 %). In total, 16,906 cases and 19,263 controls were included in the 57 studies. Table 1 shows the studies included, indicating the miRNAs and SNPs studied, the case and control sample sizes, and the continent and country where the study was conducted.

**Table 1:** International association studies between miRNA SNPs and breast cancer risk, by continent

Continent	Country/ countries	Cases	Controls	miRNA	SNP(s)	References
Africa	Tunisia	83	50	146a	rs2910164	Belaiba et al. 2018 [54]
Asia	China	321	290	499 27a 196a2 146a	rs3746444 rs895819 rs11614913 rs2910164	Qi et al. 2015 [55]
		450	450	499 149 146a 423 196a2 27a	rs3746444 rs2292832 rs2910164 rs6505162 rs11614913 rs895819	He et al. 2015 [56]
		560	583	196a2 499 608	rs11614913 rs3746444 rs4919510	Dai et al. 2016 [57]
		264	255	27a	rs895819	Zhang et al. 2013 [58]
		1009	1093	146a 149 196a2 499	rs2910164 rs2292832 rs11614913 rs3746444	Hu et al. 2009 [59]
		252	248	618 605 149 27a 196a2	rs2682818 rs2043556 rs2292832 rs895819 rs11614913	Zhang et al. 2012 [60]
		191	192	146a 373 373 27a 423 492 124-1 603 604 26a-1 605 608 100 105-1 105-2 1206 1274-a 125b-1 943 196a2 30c-1 Let-7f-2 149	rs2910164 rs12983273 rs10425222 rs895819 rs6505162 rs2289030 rs531564 rs11014002 rs2368392 rs7372209 rs2043556 rs4919510 rs1834306 rs5970293 rs5970292 rs2114358 rs318039 rs2081443 rs1077020 rs11614913 rs16827546 rs17276588 rs2292832	Ma et al. 2013 [61]
		114	189	423	rs6505162	Zhao et al. 2015 [62]
		1138	1434	608	rs4919510	Huang et al. 2012 [63]
		301	310	Let-7	rs10877887 rs13293512	Sun et al. 2019 [64]
		1064	1073	101-2	rs462480 rs1053872	Chen et al. 2014 [65]
		1064	1073	30a 30a 30a 30c-1 30c-1 30c-1 30c-2 30c-2 30d 30d	rs763354 rs852963 rs852964 rs928508 rs12743517 rs3767950 rs12208417 rs16881192 rs17709260 rs7846345	Zhou et al. 2020 [66]
	India	121	164	146a 196a2 499	rs2910164 rs11614913 rs3746444	Bansal et al. 2014 [67]
		100	100	146a 196a2	rs2910164 rs11614913	Bodal et al. 2017 [68]
	Iran	353	353	27a 196a2 146a	rs895819 rs11614913 rs2910164	Mashayekhi et al. 2018 [69]
		236	203	146a 499 196a2	rs2910164 rs3746444 rs11614913 rs185070757	Omrani et al. 2014 [70]
		100	100	499 196a2	rs3746444 rs11614913	Doulah et al. 2018 [71]
		200	200	196a2 146a	rs11614913 rs2910164	Najeti-Azar et al. 2018 [72]
		161	162	323b	rs56103835	Naderi et al. 2018 [73]
		266	288	100 124-1 218-2 301b 605 4293	rs1834306 rs531564 rs11134527 rs384262 rs2043556 rs12220909	Danesh et al. 2018 [74]
		162	180	605	rs2043556	Kazemi et al. 2020 [75]
		240	231	146a 27a	rs2910164 rs895819	Parchami Barjui et al. 2017 [76]
		100	150	196a2 499 146a	rs11614913 rs3746444 rs2910164	Afsharzadeh et al. 2017 [77]
		100	100	196a2	rs11614913	Eslami-S et al. 2018 [78]
		86	96	499	rs3746444	Kabirizadeh et al. 2016 [79]
		160	192	608	rs4919510	Hashemi et al. 2016 [80]
		129	153	599	rs58450758	Baherini et al. 2019 [81]
		129	144	520f	rs75598818	Meshkat et al. 2018 [82]
		82	70	146a	rs2910164	Meshkat et al. 2016 [83]
		263	221	34 b/c	rs4938723	Sanaei et al. 2016 [84]
		50	50	146a	rs2910164	Siasi et al. 2020 [85]
	Israel	198	290	27a	rs895819	Kontorovich 2010 [86]
	Saudi Arabia	100	100	196a2 146a 499	rs11614913 rs2910164 rs3746444	Alshatwi et al. 2012 [87]
		100	124	423	rs6505162	Mir et al. 2018 [88]
	Vietnam	106	117	27a	rs895819	Nguyen et al. 2016 [89]
		113	127	196a2	rs11614913	Minh et al. 2018 [90]
	Pakistan	300	230	146a	rs2910164	Ahmad et al. 2019 [91]
Europe	France	1130	596	146a	rs2910164	Garcia et al. 2011 [92]
	Germany	1217	1422	27a	rs895819	Yang et al. 2010 [93]
		1134	1517	196a2 499 146a	rs11614913 rs3746444 rs2910164	Catucci et al. 2010 [94]
		1217	1422	126 335	rs463297 rs41272366	Yang et al. 2011 [95]
	Ireland	523	724	146a	rs2910164	McVeigh et al. 2017 [96]
	Italy	760	1243	196a2 499 146a	rs11614913 rs3746444 rs2910164	Catucci et al. 2010 [94]
		1025	1593	27a	rs895819	Catucci et al. 2012 [97]
		81	155	146a	rs2910164	Pastrello et al. 2010 [98]
	Spain	538	189	146a	rs2910164	Cardeñosa 2012 [99]
North America	USA	441	479	196a2	rs11614913	Hoffman et al. 2009 [100]
	USA (African-American)	474	412	106b 100 331 758 544 487 659 513a-2	rs1527423 rs1834306 rs11107973 rs12586258 rs10144193 rs1951032 rs5750504 rs2018562	Yao et al. 2013 [101]
	USA (European-American)	329	310	106b 100 331 758 544 487 659 513a-2	rs1527423 rs1834306 rs11107973 rs12586258 rs10144193 rs1951032 rs5750504 rs2018562	
	USA (African-American)	894	788	185 9 − 1 9 − 2 16 − 1/15a 34b/c 206	rs2008591 rs887205 rs2078749 rs12239077 rs1501672 rs9535416 rs4938723 rs6920648 rs16882131	Bensen et al. 2013 [102]
	USA (Caucasian)	1417	1234	185 9 − 1 9 − 2 16 − 1/15a 34b/c 206	rs2008591 rs887205 rs2078749 rs12239077 rs1501672 rs9535416 rs4938723 rs6920648 rs16882131	
Oceania	Australia	173	187	145	rs353291	Chacon-Cortes et al. 2015 [103]
		193	193	423	rs6505162	Smith et al. 2012 [104]
		193	190	196a2	rs11614913	Jedlinski et al. 2011 [105]
South America	Chile	440	807	196a2 423 27a 618 608	rs11614913 rs6505162 rs895819 rs2682818 rs4919510	Morales et al. 2016 [106]
		440	1048	146a 499 125a 605 182	rs2910164 rs3746444 rs12975333 rs2043556 rs4541843	Morales et al. 2018 [107]
	Brazil	388	388	196a2	rs11614913	Linhares et al. 2012 [108]
		326	411	146a	rs2910164	Brincas et al. 2020 [109]

**Fig. 1:**
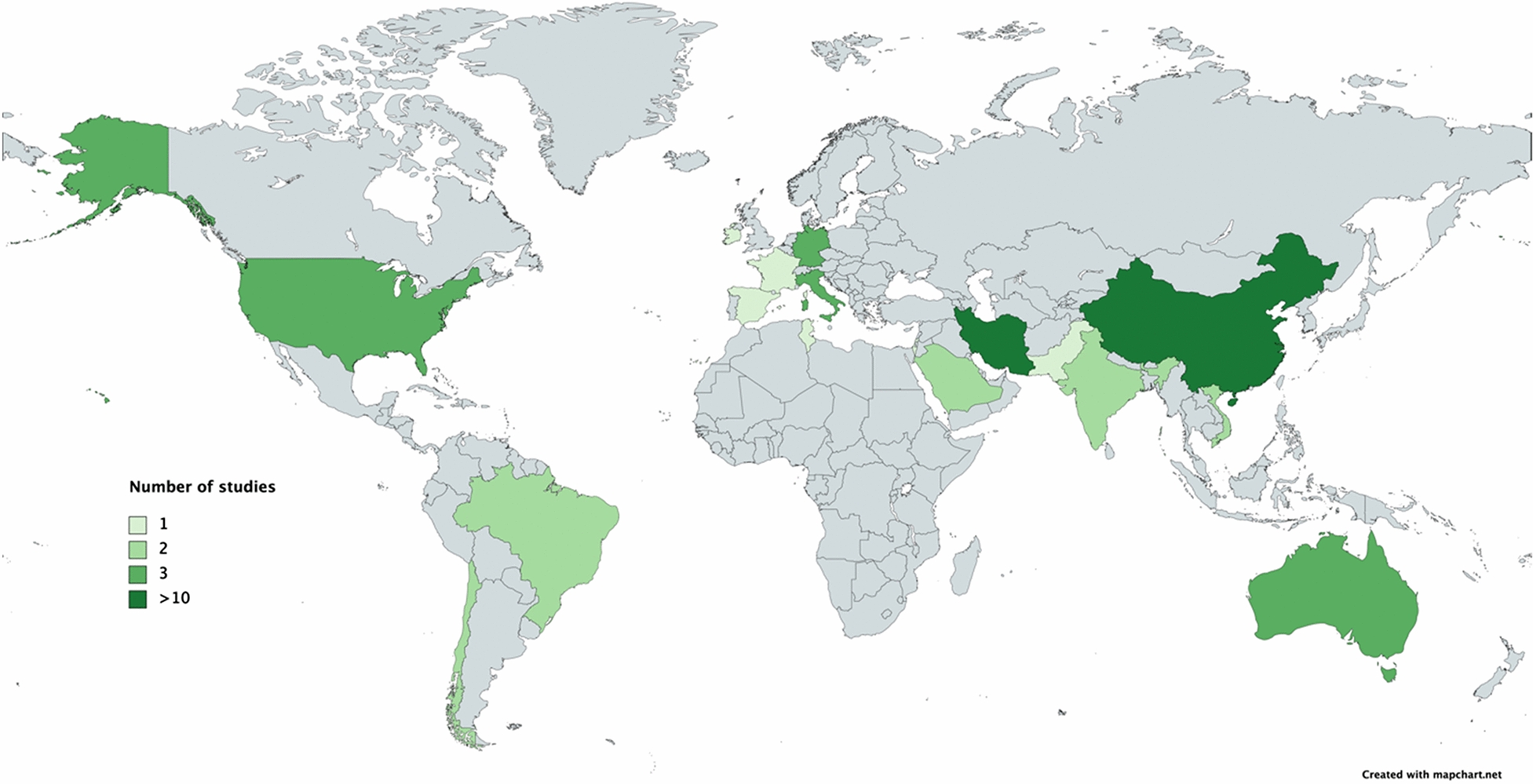
Scope of association studies between miRNA SNPs and breast cancer around the world. Green areas correspond to countries with studies included in this review. The color gradient represents the number of studies in each country

When the results were analyzed by continent, we found that Asia had the highest proportion of studies (65.0 %) and Africa the lowest (1.8 %). Within Asia, 45.9 % of the studies were conducted in Iran, 35.1 % in China, 5.4 % in Saudi Arabia, 5.4 % in India, 5.4 % in Vietnam, 2.7 % in Pakistan, and 2.7 % in Israel. The continent with the second-highest number of studies was Europe (15.8 %), where studies were carried out in 5 countries: France (11.1 %), Germany (33.3 %), Ireland (11.1 %), Italy (33.3 %), and Spain (11.1 %). Studies in South America accounted for 7.0 % of studies around the world and were performed only in Chile (50 %) and Brazil (50 %). In Oceania, studies have only been carried out in Australia, corresponding to 5.3 % of the total. In North America, the only country with association studies between miRNA SNPs and BC risk is the USA, representing 5.3 % of total studies. Finally, only one study was available for Africa, conducted in Tunisia, accounting for 1.8 % of studies worldwide. Figure 1 shows the scope of association studies between miRNA SNPs and BC risk in countries around the world.

**Fig. 2:**
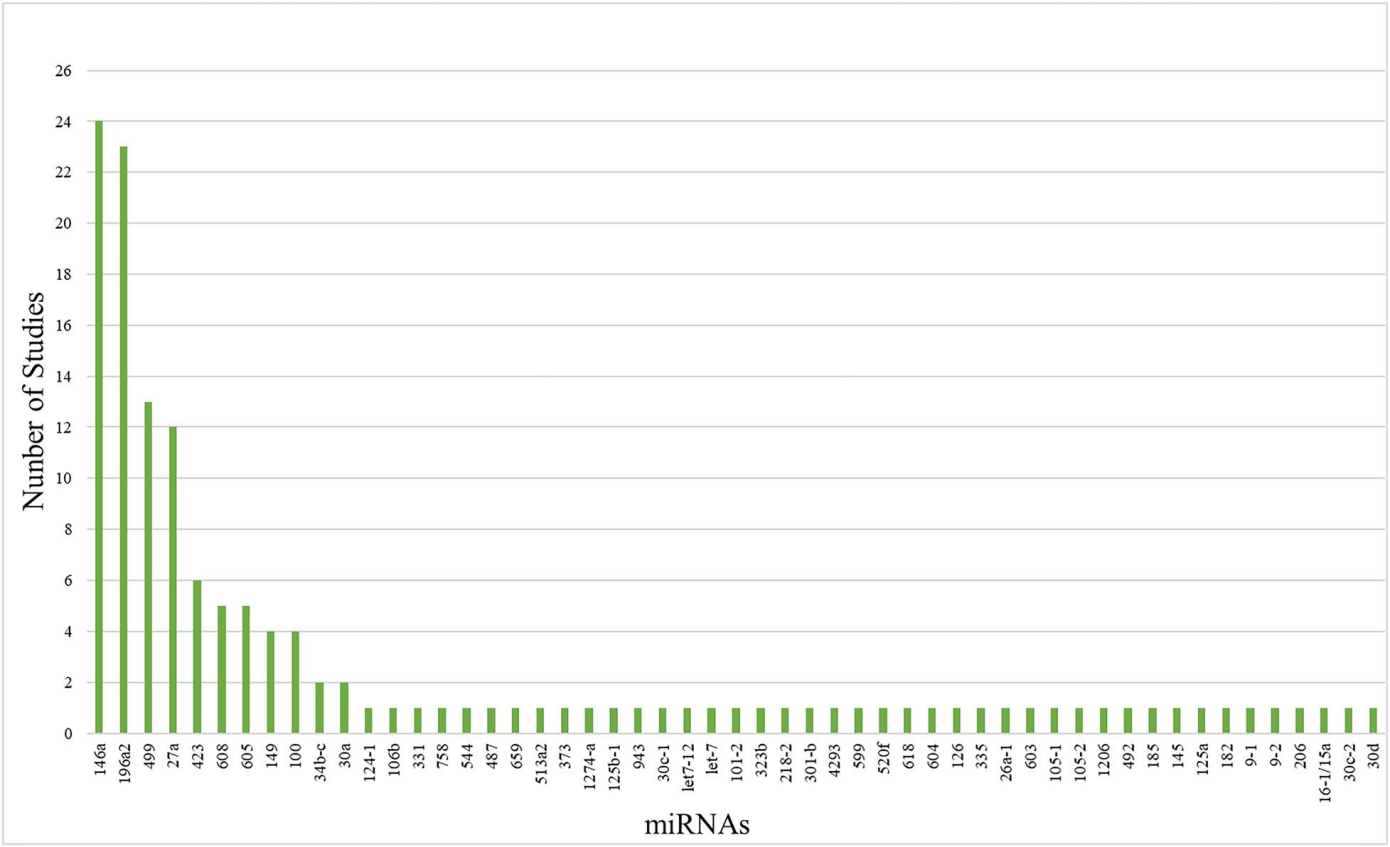
Number of studies performed for each miRNA included in this review

The SNPs studied were located in 53 different miRNAs (Table 1). Figure 2 shows the number of studies for each miRNA evaluated in this review. 146a, 196a2, 499, 27a, and 423 were the most-studied miRNAs, included at least 6 studies (Fig. 2). The most-studied miRNA was miRNA 146a, with reports from 4 to 5 continents (Africa, Asia, Europe, and America). In Africa, this miRNA only has been studied in Tunisia; in Asia, there are studies in China, India, Iran, Saudi Arabia, and Pakistan; in Europe, France, Ireland, Italy, and Spain have studies; and in the Americas, there are only studies from Chile and Brazil. The miRNA-196a2 was studied in 4 of 5 continents, (Asia, Europe, America, and Oceania). In Asia, miRNA-196a2 was studied in China, India, Iran, Saudi Arabia, and Vietnam; in Europe, in Germany and Italy; in North America, only in the USA; in South America, in Chile and Brazil; and in Oceania, only in Australia. The miRNA499 was studied in China, India, Iran, and Saudi Arabia in Asia; in Europe, there are reports from Germany and Italy; and only in Chile in South America. miRNA 27a was studied in 3 of 5 continents (Asia, Europe, and America). In Asia, studies were carried out in China, Iran, and Israel; in Europe, in Germany and Italy; and only in Chile in South America. Finally, miRNA 423 was studied in Asia, Oceania, and America. In Asia, there are reports in China and Saudi Arabia; in Oceania, in Australia; and in South America, only in Chile.

Table 2 summarizes the SNPs studied by miRNA, indicating the countries where the studies were conducted.

Sixty-seven SNPs were studied in the 53 miRNAs. In 85.0 % of the miRNAs, a single SNP was studied, and in 10.4 %, two SNPs were studied. Three different SNPs were studied in the miRNAs 185 and 30a, and 4 in the 30c-1 (Table 2). Forty different SNPs were studied in China, 14 in Iran, 18 in USA, 10 in Chile, 5 in Germany, 4 in Saudi Arabia and Italy, 3 in Australia and India, 2 in Vietnam and Brazil, and only one SNP was studied in France, Ireland, Pakistan, Spain, Tunisia, and Israel.

**Table 2:** Summary of SNPs studied by miRNA and country

miRNA	SNP(s)	Country/countries
196a2	rs11614913	Australia, Brazil, Chile, China, Germany, India, Iran, Italy, Saudi Arabia, USA, Vietnam
	rs185070757	Iran
146a	rs2910164	Brazil, Chile, China, France, India, Iran, Ireland, Italy, Pakistan, Saudi Arabia, Spain, Tunisia
499	rs3746444	Chile, China, Germany, India, Iran, Italy, Saudi Arabia
27a	rs895819	Chile, China, Germany, Iran, Israel, Italy, Vietnam
423	rs6505162	Australia, Chile, China, Saudi Arabia
608	rs4919510	Chile, China, Iran
605	rs2043556	Chile, China, Iran
149	rs2292832	China
100	rs1834306	China, Iran, USA
373	rs12983273 rs10425222	China
124-1	rs531564	China, Iran
618	rs2682818	Chile, China
106-b	rs1527423	USA
331	rs11107973	USA
758	rs12586258	USA
544	rs10144193	USA
487	rs1951032	USA
659	rs5750504	USA
513a-2	rs2018562	USA
1274a	rs318039	China
125b-1	rs2081443	China
943	rs1077020	China
30c-1	rs16827546 rs928508 rs12743517 rs3767950	China
Let7-f2	rs17276588	China
Let7	rs10877887 rs13293502	China
101-2	rs462480 rs105387	China
30a	rs763354 rs852963 rs852964	China
30c-2	rs12208417 rs16881192	China
30d	rs17709260 rs7846345	China
323b	rs56103835	China
218-2	rs11134527	Iran
301-b	rs384262	Iran
4293	rs12220909	Iran
599	rs58450758	Iran
520f	rs75598818	Iran
34b/c	rs4938723	Iran, USA
604	rs2368392	China
126	rs463297	Germany
335	rs41272366	Germany
26a-1	rs7372209	China
603	rs11014002	China
105-1	rs5970293	China
105-2	rs5970292	China
1206	rs2114358	China
492	rs2289030	China
185	rs2008591 rs887205 rs2078749	USA
145	rs353291	Australia
125a	rs12975333	Chile
182	rs4541843	Chile
9 − 1	rs12239077	USA
9 − 2	rs1501672	USA
16 − 1/15a	rs9535416	USA
206	rs6920648 rs16882131	USA

Table 3 shown the results of the association studies between miRNA SNPs and BC risk according to risk category: increased risk, protective effect, and no association. Of the 53 miRNAs and 67 SNPs included in this review, only 18 miRNAs (33.3 %) and 19 SNPs (28.4 %) (Table 3) were associated with increased risk. The Asian ethnic group had the highest number of SNPs associated with risk (16.4 %). In the USA, 6 SNPs (9 %) were associated with increased BC risk in both African- and European-American women. In South America, 5 different SNPs (7.5 %) in the miRNAs 146a, 196a2, 423, 618, and 182 were associated with increased BC risk, and in Europe (Italy) only one SNP, rs2910164 in miRNA 146a, was associated with risk (1.5 %).

Thirteen miRNAs (24.1 %) and 15 SNPs (22.4 %) were associated with decreased BC risk. In Asia, 8 SNPs (12 %) had a protective effect, and in Europe only one SNP (1.5 %) rs895819:A > G (miRNA-27a) was associated with decreased risk, in a German population. In North America (USA), 4 SNPs (6 %) located in four different miRNAs were associated with decreased BC risk in African- and European-American women. In South America, 3 SNPs (4.5 %) were protective, and in Oceania (Australia), the only SNP associated with decreased BC was rs6505162:A > C in miRNA-423 (1.5 %). Of the total miRNAs included in this review, 28 different miRNAs (52.8 %) and 31 different SNPs (46.3 %) were associated with BC risk.

Genetic variations are ethnicity-specific; therefore, results of association studies between a miRNA SNP and BC risk may diverge depending on ethnicity. The most-studied SNP, rs2910164:G > C in miRNA-146a, was found to increase the risk of developing breast cancer in Brazilian, Chinese, Iranian, Italian, and Pakistani populations (Table 3) but showed no association in Chilean, Chinese, French, German, Iranian, Irish, Italian, Saudi Arabian, Spanish, or Tunisian populations (Table 3). The heterozygous variant showed a protective effect in a North Indian population (Table 3). With respect to the second most-common SNP, rs11614913:C > T in miRNA-196a2 was associated with increased risk in Brazilian, Chinese, Iranian, and Saudi Arabian populations but showed no association in Chilean, Caucasian Australian, Chinese, Iranian, Indian, Italian, or German populations and was protective in Brazil, China, USA, and Vietnam. The studies in China were conducted in different regions of the country, and the Brazilian study by Linhares et al. (2012) [108], showed that for the SNP rs11614913:C > T, the allele T increased risk, while the allele C had a protective effect (Table 4). The SNP rs3746444:A > G in miRNA-499 increased risk in Chinese, Iranian and Saudi Arabian populations, and showed a protective effect in an Iranian population (genotype CC and CT) (Table 4), but showed no association in Chilean, Chinese, German, North Indian, or Italian populations. The rs895819:A > G in miRNA-27a was protective in Chilean, Chinese, German, Iranian, and Israeli populations, but showed no association in Chinese, Italian, or Vietnamese populations. Another relatively common SNP was rs6505162, located in miRNA-423. This SNP showed an association with increased BC risk in Chilean and Saudi Arabian populations but had a protective effect in a Caucasian Australian population.

**Table 3:** Association categories for miRNA SNPs and breast cancer risk

BC risk category	miRNA	SNP(s)	Country/countries	Continent(s)
Increased risk	146a	rs2910164:G > C	Brazil, China, Iran, Italy, Pakistan	Asia, Europe, South America
	196a2	rs11614913:C > T	Brazil, China, India, Iran, Saudi Arabia	Asia, South America
	499	rs3746444:T > C	China, Iran, Saudi Arabia	Asia
	218-2	rs11134527:A > G	Iran	Asia
	301-b	rs384262G > A	Iran	Asia
	605	rs2043556T:A > G	Iran	Asia
	599	rs58450758:C > T	Iran	Asia
	423	rs6505162:C > A	Chile and Saudi Arabia	Asia, South America
	513a-2	rs2018562	USA (African-American)	North America
	106b	rs1527423:A > G	USA (European-American)	North America
	182	rs4541843:C > T	Chile	South America
	101-2	rs462480:A > C rs105387:C > G	China	Asia
	Let-7	rs13293512:T > C	China	Asia
	331	rs1110793:A > G	USA (European-American)	North America
	544	rs10144193:A > T	USA (European-American)	North America
	487	rs1951032G > A	USA (European-American)	North America
	659	rs5750504:T > A	USA (European-American)	North America
	618	rs2682818C > A	Chile (early-onset BC)	South America
Decreased risk	27a	rs895819:A > G	Chile, China, Germany, Iran, Israel	Asia, Europe, South America
	499	rs3746444:T > C	Iran	Asia
	608	rs4919510:C > G	Iran	Asia
	520f	rs75598818:G > A	Iran	Asia
	196a2	rs11614913:C > T	Brazil, China, USA, Vietnam	Asia, North America, South America
	758	rs12586258:G > A	USA (African-American)	North America
	100	rs1834306:G > A	USA (European-American)	North America
	185	rs2008595:C > T rs887205:A > G	USA (African-American)	North America
	423	rs6505162:A > C	Australia	Oceania
	605	rs2043556:T > C	Chile	South America
	146a	rs2910164:G > C	India	Asia
	149	rs2292832:T > C	China	Asia
	30a	rs763354:G > A	China	Asia
No association	196a2	rs11614913:C > T	Australia, Chile, China, Germany, Iran, India, Italy	Asia, Europe, South America, Oceania
	196a2	rs185070757	Iran	Asia
	146a	rs2910164:G > C	Chile, China, France, Germany, Iran, Ireland, Italy, Saudi Arabia, Spain, Tunisia	Africa, Asia, Europe, South America
	323b	rs56103835:T > C	Iran	Asia
	100	rs1834306:T > C	China, Iran, USA (African-American)	Asia, North America
	124-1	rs531564:G > C	China, Iran	Asia
	605	rs2043556:T > C	China, Iran	Asia
	4293	rs12220909:G > C	Iran	Asia
	34b/c	rs4938723:	Iran	Asia
	27a	rs895819:A > C	China, Italy, Vietnam	Asia and Europe
	499	rs3746444:T > C	Chile, China, Germany, India, Italy	Asia, Europe, South America
	126	rs463297	Germany	Europe
	335	rs41272366	Germany	Europe
	106b	rs1527423:A > G	USA (African-American)	North America
	331	rs11107973:A > G	USA (African-American)	North America
	758	rs12586258:G > A	USA (African-American)	North America
	513a-2	rs2018563:A > G	USA (African-American)	North America
	185	rs2078749:A > G	USA (African-American)	North America
	145	rs353291:T > C	Australia	Oceania
	608	rs4919510:C > G	Chile, China	Asia, South America
	125a	rs12975333:A > G	Chile	South America
	423	rs6505162:C > A	China	Asia
	149	rs2292832:T > C	China	Asia
	9−1	rs12239077:A > G	USA (African- and European-American)	North America
	9−2	rs1501672:T > C	USA (African- and European-American)	North America
	16−1/15a	rs9535416:G > A	USA (African- and European-American)	North America
	34b/c	rs4938723:T > C	USA (European-American)	North America
	206	rs6920648:A > G rs16882131:C > T	USA (European-American)	North America
	185	rs28591:C > T rs887205:A > G rs2078749:A > G	USA (European-American)	North America
	618	rs2682818:C > A	China	Asia
	373	rs12983273:C > T rs1042522:C > A	China	Asia
	492	rs2289030:C > G	China	Asia
	603	rs11014002:C > T	China	Asia
	604	rs2368392:C > T	China	Asia
	26a-1	rs7372209:C > T	China	Asia
	105-1	rs5970293:G > C	China	Asia
	105-2	rs5970292:G > A	China	Asia
	1206	rs2114358:T > C	China	Asia
	1274a	rs318039:C > T	China	Asia
	125b-1	rs2081443:T > G	China	Asia
	943	rs1077020:C > T	China	Asia
	30c-1	rs16827546:C > T rs928508:A > G rs12743517:C > A rs3767950:C > A	China	Asia
	Let-7f-2	rs17276588:G > A	China	Asia
	Let-7	rs10877887:T > C	China	Asia
	30a	rs852963:G > A rs852964:G > A	China	Asia
	30c-2	rs12208417:C > A rs16881192:A > C	China	Asia
	30d	rs17709260:A > G rs7846345:G > C	China	Asia

Table 4 shows the allele or genotype associated with BC risk in the miRNA SNPs included in this review. For the most-studied SNPs, which were analyzed in at least 6 studies, controversial results are observed. For the miRNA-146a rs2910164:G > C, the C allele was the MAF and risk allele in Italy, Pakistan, Iran, and Brazil; in China, however, the risk genotypes were CG and homozygous GG, and allele G was the MAF and risk allele. For the miRNA-196a2 rs11614913:C > T, the risk allele was C in two studies from China as well as studies from Iran and India. Nevertheless, in Brazil and Saudi Arabia, two ethnically-different countries, the risk allele was T. Other discrepancies are shown in Table 4. In sum, association results from a single study should be interpreted and analyzed with caution. Factors to consider include cohort size, ethnicity, and ancestral lineage, especially in countries with more than one lineage.

**Table 4:** Allele or genotype associated with breast cancer risk in miRNA SNPs included in this review

BC risk category	miRNA	SNP(s)	Risk allele or genotype	*p-*value	Countries	References
Increased risk	146a	rs2910164:G > C	C CG and GG C CC C C GC and CC CC	0.03 < 0.05 0.04 < 0.001 0.03 0.0037 0.033 and 0.028 < 0.0001	Brazil China China Iran Iran Iran Italy Pakistan	Brincas et al. 2020 [109] He et al. 2015 [56] Qi et al. 2015 [55] Mashayekhi et al. 2018 [69] Parchami Barjui et al. 2017 [76] Meshkat et al. 2016 [83] Pastrello et al. 2010 [98] Ahmad et al. 2019 [91]
	196a2	rs11614913:C > T	T CT C C C CT	0.024 0.04 0.01 0.011 0.02236 0.01	Brazil India China China Iran Saudi Arabia	Linhares et al. 2012 [108] Bodal et al. 2017 [68] Qi et al. 2015 [55] Hu et al. 2009 [59] Najeti-Azar et al., 2018 [72] Alshtawi et al. 2012 [87]
	499	rs3746444:T > C or A > G	AG and GG G C C G C	0.008 0.025 0.001 0.034 0.02952 0.001	China China Iran Iran Iran Saudi Arabia	Dai et al. 2016 [57] Hu et al. 2009 [59] Omrani et al. 2014 [70] Afsharzadeh et al., 2017 [77] Kabirizadeh et al., 2016 [79] Alshtawi et al. 2012 [87]
	218-2	rs11134527:A > G	G	< 0.0001	Iran	Danesh et al. 2018 [74]
	301-b	rs384262G > A	A	< 0.0001	Iran	Danesh et al. 2018 [74]
	605	rs2043556T:A > G	G	0.00003	Iran	Kazemi et al. 2020 [75]
	599	rs58450758:C > T	CT and TT	< 0.0001	Iran	Bahreini et al. 2019 [81]
	423	rs6505162:C > A	A T	0.02 0.0001	Chile Saudi Arabia	Morales et al. 2016 [106] MiR et al. 2018 [88]
	513a-2	rs2018562:A > G	G	0.03	USA	Yao et al. 2013 [101]
	106b	rs1527423:A > G	G	0.02	USA	Yao et al. 2013 [101]
	182	rs4541843:C > T	T	0.01	Chile	Morales et al. 2018 [107]
	101-2	rs462480:A > C rs105387:C > G	C G	0.017 0.010	China	Chen et al. 2014 [65]
	Let-7	rs13293512:T > C	C	0.013	China	Sun et al., 2019 [64]
	331	rs1110793:A > G	G	0.02	USA	Yao et al. 2013 [101]
	544	rs10144193:A > T	T	0.004	USA	Yao et al. 2013 [101]
	487	rs1951032G > A	A	0.001	USA	Yao et al. 2013 [101]
	659	rs5750504:T > A	A	0.03	USA	Yao et al. 2013 [101]
	618	rs2682818C > A	CA	0.03	Chile	Morales et al. 2016 [106]
Decreased risk	27a	rs895819:A > G or T > C	GG G G G G T	0.01 0.032 0.0287 < 0.001 0.001 0.013	Chile China Germany Iran Iran Israel	Morales et al. 2016 [106] Zhang et al. 2013 [58] Yang et al. 2010 [93] Mashayekhi et al. 2018 [69] Parchami Barjui et al. 2017 [76] Kontorovich et al. 2010 [86]
	499	rs3746444:T > C	C	0.003	Iran	Doulah et al. 2018 [71]
	608	rs4919510:C > G	G	0.024	Iran	Hashemi et al. 2016 [80]
	520f	rs75598818:G > A	GA	0.041	Iran	Meshkat et al. 2018 [82]
	196a2	rs11614913:C > T	CC T T T	0.009 0.0005 0.002 0.00295	Brazil China USA Vietnam	Linhares et al. 2012 [108] Dai et al. 2016 [57] Hoffman et al. 2009 [100] Mihn et al. 2018 [90]
	758	rs12586258:G > A	A	0.01	USA	Yao et al. 2013 [101]
	100	rs1834306:G > A	A	0.02	USA	Yao et al. 2013 [101]
	185	rs2008595:C > T rs887205:A > G	TT GG	0.04 0.03	USA	Bensen et al. 2013 [102]
	423	rs6505162:A > C	CC	0.035	Australia	Smith et al. 2012 [104]
	605	rs2043556:T > C	C	0.02	Chile	Morales et al. 2018 [107]
	146a	rs2910164:G > C	C	0.01	India	Bansal et al. 2014 [67]
	149	rs2292832:T > C	CC	0.053	China	He et al. 2015 [56]
	30a	rs763354:G > A	A	0.022	China	Zhou et al. 2020 [66]

## Discussion

The majority of the association studies between miRNA SNPs and BC risk were carried out in Asia. To paint a more complete view of the influence of miRNA SNPs on BC risk, it will therefore be necessary to perform this type of study in more American, Oceanic, and African countries. Most of the studies had small sample sizes, which, as is well known, may influence the precision of the results and the power of the studies to draw conclusions. Although multiple meta-analyses in recent years have attempted to define the association between certain miRNA polymorphisms and BC risk more precisely, there still seems to be no clear consensus. It has been established that SNPs are the most common source of variability in the human genome and that these variations are ethnicity-specific. Thus, the effect of a specific SNP on BC risk may differ depending of the ethnicity of a specific population. Chen, Q. et al. 2014, observed that miR-196a-2 rs11614913*T, miR-499 rs3746444*T, and miR-605 rs2043556*A alleles predicted a decreased risk of breast cancer among Asians but not Caucasians [19]. Fejerman et al. [110], performed a study comparing genetic variants in Hispanic and non-Hispanic white women based on the fact that Hispanic women in the USA have been shown to have a lower incidence of BC [110]. The authors observed that 3 of 5 variants were associated with BC risk in Hispanic women but not in non-Hispanic women and suggested that the proportion of indigenous American ancestry modified the magnitude and direction of risk associations in 3 of the 10 variants studied. Therefore, the authors concluded that genetic ancestry is a factor to consider when performing association studies in women of mixed descent [110].

Controversial results were observed for the most-studied SNPs, each analyzed in at least 6 studies: miRNA-146a rs2910164:G > C, miRNA-192a2 rs11614913:C > T, miRNA-499 rs3746444:T > C, and miRNA-27a rs895819:A > G.

The miRNA-146a rs2910164 showed controversial results in China, Iran, and Italy. Four studies performed in China included this SNP. The SNP was associated with increased risk in two of these studies [55, 56], but not in the other two [59, 61]. In the articles by He et al. [56], Qi et al. [55], and Ma et al. [61], the case and control sample sizes were small (Table 1). In the Hu et al. [59], study, which included 1009 cases and 1093 controls, rs2910164:G > C was not associated with BC risk. China is a country with many different ethnicities. The study by Qi et al. [55], was conducted in Henan province, which is widely recognized as the place where Chinese civilization originated. In this study, the SNP was associated with increased BC risk. The other 3 studies were conducted in the same region or nearby provinces. However, the discrepancies between these studies may be due to the fact that He et al. identified the risk association in a sample of postmenopausal women with BC, while Qi et al. [55], simply indicated that there was an increased BC risk without further specifications. Regarding the two studies that did not find an association, the Ma et al. study assessed a sample of women with triple-negative BC, while Hu et al. [59], indicated no association with BC risk without further specifications. Therefore, the divergent results for this SNP in the populations studied could be a consequence of the characteristics of the cases.

miRNA-192a2 rs11614913:C > T showed controversial results in Brazil, China, and Iran. In Brazil, Linhares et al. [108], reported an increased risk for the T-allele but a protective effect for the wild-type CC genotype, which is not a discrepancy. In China, 5 authors studied this SNP. In two publications, the SNP was associated with increased BC risk [55, 56]; in one study it was associated with decreased risk [57]; and in two studies there was no association between the SNP and risk [60, 61]. These findings could be the consequence of ethnic differences. The Hu et al. [59], study included a Nanjing population, where the main ethnic group is Han, but 50 other official ethnic groups are also present. In this population, the SNP was associated with increased BC risk. In the Dai et al. study [57], in which the SNP was associated with decreased BC risk, the ethnicity of the population studied was mainly Han. Zhang et al. found no association between the SNP and BC risk in a population from Zhejiang province, where the ethnic groups include Han, She, Hu, and 49 minority ethnic groups. All of the authors who studied rs11614913 used cases with BC without specifying whether the cancer was familial, sporadic, or early-onset or had other notable characteristics. Consequently, the heterogeneity of the types of BC included in the samples could provoke discrepancies in the results. It is likely that divergent results from Iranian studies are fundamentally due to variations in ethnicity, as this country includes Persians (the main ethnic group), Azeris, Kurds, Lurs, Turkmens, and Baloch, and others.

The discrepancies observed for miRNA-499 rs3746444:T > C and miRNA-27a rs895819:A > G from studies conducted in China and Iran can be explained by the same reasons discussed for miRNA-146a and miRNA-196a2.

It is clear that more studies in Western populations are needed. In South America, only two countries, Chile and Brazil, have performed such studies. This situation underrepresents Western populations. Another issue that all of these studies classified Asians as a single population group despite the fact that Asia is extremely diverse. It was recently reported that the continent has at least ten ancestral lineages, while areas such as northern Europe have only one [111]. In this review, we included studies from China, Iran, India, Saudi Arabia, Israel, and Vietnam, countries with very different ethnicities and genetic profiles. Unfortunately, these differences are not considered in most population-based analyses. The GenomeAsia100k consortium has addressed this problem, noting that underrepresentation of non-Europeans in genetic studies has limited the diversity of individuals in genomic datasets. As a result, many findings have limited medical relevance for a large proportion of the world’s population [112]. The need for more specific population-based studies is clear, with Asian populations separated into more homogenous groups.

In a clinical context, molecular information regarding breast cancer has become highly relevant. The World Health Organization emphasizes that early diagnosis of BC is critical for optimizing outcomes and survival [113]. Unfortunately, the available molecular diagnostic methods may pose limitations. Therefore, miRNAs have emerged as possible diagnostic and prognostic biomarkers. miRNAs also have a potential role in personalized therapy [114]. Srinivasan et al. 2016, has reported that SNPs are more precise genetic determinants than family history; furthermore, SNP genotyping can be performed without the need for invasive techniques [115].

## Conclusions

This review examined the sometimes-conflicting results available in the international literature regarding the impact of miRNA polymorphisms on BC risk. We can conclude that: (a) The greatest proportion of studies on this topic have been carried out in Asia (65.0 %), while only one such study has been performed in Africa (1.8 %). In South America, studies have only been conducted in Chile (50 %) and Brazil (50 %), and in Oceania, studies have only been carried out only in Australia; (b) Association studies have been performed for 67 SNPs, located in 53 miRNAs; (c) 146a, 196a2, 499, 27a, and 423 are the most-studied miRNAs, with each included in at least 6 studies; (d) Most of the studies had small samples, possibly limiting the precision of the results and the power to draw conclusions; and (e) This review demonstrates that the effect of a specific SNP on BC risk varies according to the ethnicity the population. It is crucial that comprehensive evaluations be performed in larger cohorts, stratified by ethnicity and histological subtype, to better define the associations between miRNA polymorphisms and BC risk.

## Data Availability

All data are shown within the manuscript.
